# A Case Series of Late Myocardial Infarction Following Self-Expanding Transcatheter Aortic Valve Replacement

**DOI:** 10.14740/jmc5303

**Published:** 2026-04-29

**Authors:** Muhammad Saad, Mohammad Hashim Jilani, Mohsin Munawar, Cristiano Spadaccio, Rishi Sukhija, Arif Imran

**Affiliations:** a Department of Internal Medicine, The Jewish Hospital of Cincinnati, Cincinnati, OH, USA; b Division of Cardiology, University of Cincinnati School of Medicine, Cincinnati, OH, USA; c Department of Cardiothoracic Surgery, University of Cincinnati School of Medicine, Cincinnati, OH, USA

**Keywords:** TAVR, Acute myocardial infarction, Aortic stenosis, Transcatheter, Aspiration thrombectomy

## Abstract

Embolic stroke is a recognized complication of transcatheter aortic valve replacement (TAVR); however, coronary embolism is rarely reported, particularly when occurring late after valve implantation. We described three patients presenting with ST-segment elevation myocardial infarction (STEMI) between 10 and 50 months after self-expanding TAVR. All patients had angiographically normal coronary arteries prior to TAVR, severe native aortic valve calcification, and well-controlled cardiovascular risk factors, and were maintained on guideline-directed single antiplatelet therapy. At presentation, coronary angiography demonstrated abrupt 100% coronary occlusion without angiographic evidence of underlying atherosclerotic disease. Aspiration thrombectomy was required in all three cases. These cases illustrate a rare presentation of late myocardial infarction following self-expanding TAVR with angiographic features possibly suggestive of a non-atherosclerotic mechanism. Although a definitive embolic source cannot be established, delayed embolization of calcific or thrombotic material may represent a plausible explanation. These observations are hypothesis-generating and underscore the need for clinical vigilance and further mechanistic and imaging-based studies to better characterize potential late thromboembolic pathways after TAVR.

## Introduction

Aortic stenosis (AS) is one of the most prevalent valvular heart diseases [1] with a high mortality rate of up to 50% at 2 years in symptomatic patients [2]. Aortic valve replacement, either transcatheter aortic valve replacement (TAVR) or surgical aortic valve replacement (SAVR), is the treatment of choice for severe symptomatic AS.

TAVR is becoming increasingly common, supported by trials such as PARTNER 3 and EVOLUT, which have demonstrated that TAVR is either superior or at least non-inferior to SAVR [3]. Initially, TAVR was reserved for high-risk patients who were deemed unsafe for surgical replacement, but now it is approved for aortic valve replacement in patients with symptomatic AS, including those at high, intermediate, or low risk [4]. In TAVR, two types of valves are widely used: self-expanding valves (SEVs) and balloon-expandable valves (BEVs) [5]. Paravalvular leak (PVL), conduction disturbances, and stroke are some well-known complications of TAVR.

Systemic embolism, causing stroke (1–2% incidence), is a well-documented early periprocedural complication. According to current American and European guidelines, lifelong single antiplatelet therapy (SAPT) with aspirin is recommended after TAVR unless there is another indication for dual antiplatelet therapy (DAPT) or anticoagulation [6, 7].

We present a case series of three patients who developed late ST-segment elevation myocardial infarction (STEMI) due to abrupt coronary occlusion several months to years after self-expanding TAVR, highlighting a rare clinical phenotype and raising hypotheses regarding potential mechanisms.

## Case Reports

### Case 1

A 66-year-old man with severe bicuspid AS underwent TAVR with a 34-mm self-expanding Evolut PRO+ valve. The native aortic valve was severely calcified with an Agatston calcium score of 2,443. Balloon valvuloplasty and post-dilation were performed during the TAVR procedure. The patient was discharged on lifelong SAPT with aspirin.

**Figure 1:**
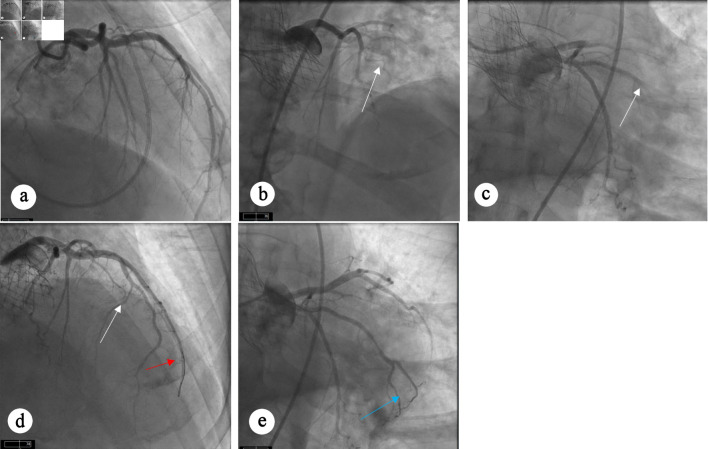
Angiographic views showing coronary arteries. (a) Diagnostic angiogram before TAVR procedure showing angiographically normal coronary arteries without any evidence of obstructive CAD. (b) Occlusion of LAD and diagonal branch artery. (c) Occlusion of OM artery. (d, e) Restoration of flow can be seen in LAD (white arrow), diagonal branch (red arrow), and OM artery (blue arrow) after aspiration thrombectomy. CAD: coronary artery disease; LAD: left anterior descending; OM: obtuse marginal; TAVR: transcatheter aortic valve replacement.

Fifty months after TAVR, he presented with anterolateral STEMI. Notably, the pre-TAVR coronary angiogram demonstrated normal coronary arteries without evidence of obstructive coronary artery disease (CAD) (Fig. 1a); however, repeat left heart catheterization at the time of presentation with STEMI revealed abrupt 100% occlusion of the left anterior descending artery (Fig. 1b), diagonal branch (Fig. 1b), and obtuse marginal (OM) branch (Fig. 1c), consistent with an embolic pattern. Aspiration thrombectomy restored TIMI 3 flow in all the affected vessels (Fig. 1d, e).

### Case 2

A 79-year-old man with severe trileaflet AS underwent TAVR using a 29-mm Evolut FX valve. Native valve calcium score was 2,972. Balloon valvuloplasty was performed without post-dilation. He received lifelong aspirin monotherapy.

**Figure 2:**
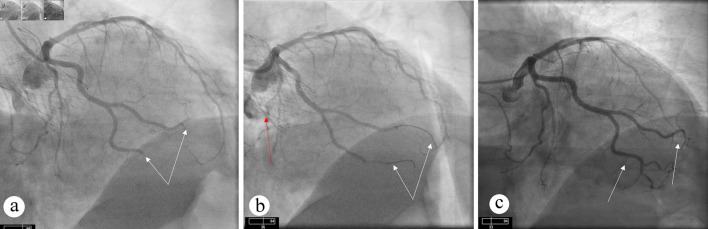
Angiographic views showing occlusion and restoration of flow after intervention. (a) Occlusion of OM branches (white arrows) can be seen. (b) Restoration of flow can be seen in OM branches (white arrow) after aspiration thrombectomy. TAVR valve (red arrow) can also be appreciated. (c) Diagnostic angiogram before TAVR procedure showing patent OM branches. OM: obtuse marginal; TAVR: transcatheter aortic valve replacement.

Ten months after TAVR, the patient presented with inferolateral STEMI. Coronary angiography demonstrated abrupt 100% occlusion of the two OM branches (Fig. 2a). Aspiration thrombectomy resulted in restoration of TIMI 3 flow in both OM branches (Fig. 2b). Review of pre-TAVR coronary angiogram confirmed previously patent vessels without any evidence of obstructive CAD (Fig. 2c).

### Case 3

An 80-year-old man with severe trileaflet AS underwent TAVR with a 29-mm Evolut PRO+ valve. The native valve calcium score was 3,422, and no balloon valvuloplasty or post-dilation was performed during the TAVR procedure. The patient was discharged on aspirin monotherapy.

**Figure 3:**
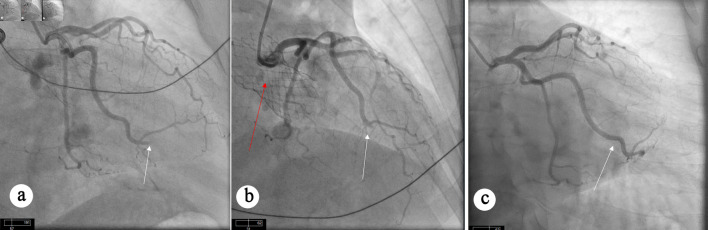
Angiographic views showing persistent occlusion of OM branch. (a) Occlusion of OM artery (white arrow) can be seen. (b) Persistent occlusion of OM artery (white arrow) despite aspiration thrombectomy. TAVR valve (red arrow) can also be seen. (c) Diagnostic angiogram before TAVR procedure showing patent OM artery without obstructive CAD. CAD: coronary artery disease; OM: obtuse marginal; TAVR: transcatheter aortic valve replacement.

Twenty-nine months later, he presented with inferolateral STEMI. Coronary angiography showed complete occlusion of an OM branch (Fig. 3a). Despite multiple attempts with balloon angioplasty and aspiration thrombectomy, adequate coronary flow could not be restored (Fig. 3b). Notably, the pre-TAVR coronary angiogram demonstrated patent OM branches without any evidence of obstructive CAD (Fig. 3c).

## Discussion

STEMI following TAVR is associated with worse clinical outcomes and higher short- and long-term mortality, likely attributable to the advanced comorbidity burden of this patient population [8, 9]. Although non-atherothrombotic mechanisms such as coronary embolism have been described in the literature [8, 10, 11], their precise contribution and clinical significance in TAVR patients remain incompletely understood. Our case series highlights a distinct angiographic pattern of late STEMI occurring in patients without prior obstructive CAD, characterized by abrupt coronary occlusion requiring aspiration thrombectomy in the setting of minimal atherosclerotic plaque. These features raise the possibility of an embolic mechanism. Supporting this hypothesis, a recent case report documented histopathologically confirmed calcific coronary embolization as a cause of STEMI following TAVR; however, this finding was associated with the ACURATE neo2 M valve [12].

Several mechanisms may plausibly account for these findings, including delayed embolization of calcific debris from severely calcified native valves, subclinical leaflet thrombosis, or valve-related flow disturbances. Importantly, these events occurred despite adherence to guideline-directed SAPT and should not be interpreted as justification for escalation of routine antithrombotic therapy, which is contraindicated in current European guidelines [7]. Alternative explanations cannot be completely excluded. Plaque erosion, occult atrial arrhythmias, or *in-situ* coronary thrombosis remain possible given the absence of intravascular imaging, histopathologic analysis, or contemporary cardiac computed tomography. Accordingly, these observations should be interpreted as hypothesis-generating rather than evidence of a causal relationship between TAVR and late coronary embolic events.

This case series has several limitations, including a small sample size, retrospective design, and lack of advanced imaging or histopathologic confirmation of embolic material. Incomplete procedural anatomic data, such as coronary heights and valve implantation depth, further limit mechanistic inference.

Taken together, STEMI occurring late after self-expanding TAVR appears to be uncommon but associated with worse outcomes [8]. It may plausibly reflect a non-atherosclerotic process in selected patients with low traditional cardiovascular risks and angiographic findings not suggestive of plaque rupture. Intravascular imaging, rhythm monitoring, and histopathological and mechanistic studies are needed to better characterize the underlying etiology of STEMI in this population and potentially inform management strategies.

### Conclusions

Late myocardial infarction after TAVR is rare but, in certain patients, may plausibly represent a non-atherosclerotic process occurring despite adherence to guideline-directed antiplatelet therapy. In patients with low baseline atherosclerotic risk factors, absence of atrial arrhythmias or intracardiac thrombus, and coronary angiography demonstrating abrupt vessel occlusion without underlying atherosclerotic disease, an embolic mechanism may be considered as a potential explanation. Intravascular imaging and histopathological evaluation, when feasible, may provide valuable insights into lesion characterization. Severe native aortic valve calcification may represent a theoretical substrate for delayed embolic phenomena after TAVR; however, a direct causal relationship cannot be established based on the current observations. These findings are exploratory and hypothesis-generating and should not be interpreted as evidence to modify current guideline-recommended antithrombotic strategies.

## Data Availability

The authors declare that data supporting the findings of this study are available within the article. Additional information is available from the corresponding author upon reasonable request.

## Abbreviations

AS: aortic stenosis
BEV: balloon expandable valve
CAD: coronary artery disease
DAPT: dual antiplatelet therapy
LAD: left anterior descending
MI: myocardial infarction
OM: obtuse marginal
PVL: paravalvular leak
SAPT: single antiplatelet therapy
SAVR: surgical aortic valve replacement
SEV: self-expanding valve
STEMI: ST-segment elevation myocardial infarction
TAVR: transcatheter aortic valve replacement
